# Pediatric palliative care in Brazil: reflections on end of life based on geographic mapping

**DOI:** 10.1007/s43999-024-00054-w

**Published:** 2024-11-28

**Authors:** Esther Angélica Luiz Ferreira, Leandro Saito, Maycon Rodrigo Sarracini, Cristina Helena Bruno, Augustus Relo Mattos, Cristina Ortiz Sobrinho Valete, Rodrigo Bezerra de Menezes Reiff

**Affiliations:** 1 Department of Medicine at the Federal University of São Carlos (DMed UFSCar), São Carlos, SP Brazil; 2 Center for Studies in Pain and Palliative Care, Federal University of São Carlos, São Carlos, SP Brazil; 3 Department of Physical Education and Human Motricity, Federal University of São Carlos, São Carlos, SP Brazil

**Keywords:** Palliative Care, Palliative Medicine, Maternal-child Health services, Pediatrics, Geographic Mapping, Atlas, End-of-life

## Abstract

**Introduction:**

In Brazil, a country of continental dimensions, the lack of services in the different regions is a major barrier that prevents patients from accessing Pediatric Palliative Care (PPC). If accessing PPC is already challenging, end-of-life care for these patients may also be difficult. Therefore, this study is based on a recent mapping effort, aimed at reflecting on the end-of-life care for children in Palliative Care in Brazil.

**Method:**

Descriptive, cross-sectional study, and online survey research, based on a larger study of Palliative Care Network.

**Results:**

The final sample comprised 90 Pediatric Palliative Care services, which proved to be unevenly distributed across the country. Many services lack a minimum team, 40% face difficulties accessing opioids, and one-third do not provide bereavement care.

**Discussion:**

There should be more services with better distribution across the country, and a minimum team should be required to provide adequate care for children and adolescents. Strategies to improve symptom control and grief support should be introduced.

**Conclusions:**

It is concluded that continuing education and the inclusion of relevant topics in health courses are necessary. Additionally, health service managers must expand their focus to address these issues effectively.

**Supplementary Information:**

The online version contains supplementary material available at 10.1007/s43999-024-00054-w.

## Introduction

Palliative Care can be understood as care aimed at improving the quality of life for patients and their families facing a serious, life-threatening illness, without excluding curative treatment [1]. In pediatrics, the profile of pediatric patients has been changing recently, due to technological advances in health and the increasing severity of conditions in children who survive complex diseases. The demands for assistance from these children, who live with chronic and potentially fatal diseases, are becoming increasingly frequent [1].

In this context, Pediatric Palliative Care (PPC) has emerged as a form of comprehensive care for these patients and their families. PPC can alleviate symptoms by addressing multidimensional aspects, such as psychological and spiritual aspects, while treating death as a natural process. This approach helps family members cope with grief and aims to strengthen the patient’s support network, assisting them in adapting to new situations caused by their condition [1]. In PPC, children require “early identification and assessment, in addition to adequate treatment, to improve quality of life, promote dignity and comfort without accelerating or delaying death, and may even positively influence the course of the disease, an essential aspect for the prognosis of pediatric patients” [2].

Regarding the end of life, “End of Life Care” is an important component of Palliative Care. It refers to the assistance a patient should receive during the final stage of their life, from the moment it becomes clear that they are in a state of progressive and inexorable decline, approaching death. Care at this stage must be impeccable to alleviate the individual’s suffering. Both Palliative Care and PPC services need to be attentive to fully meet this demand [3].

In the case of hospital care, it is essential for the facility to have adequate physical space, which should include not only beds and consultation rooms but also areas for private conversations with family members and for interdisciplinary team discussions. Regarding PPC, there are specific considerations due to the ongoing physical, hormonal, cognitive, expressive, and emotional development of children. This complexity requires a specialized and experienced pediatric care team [1].

Although countless patients could benefit from PPC, access to these services still presents many difficulties [4]. The management of PPC services is a critical issue but remains underdeveloped in Latin American, Asian and African countries compared to North American and European countries [5].

In Brazil, a country of continental dimensions, the lack of services across different regions is a major barrier that prevents patients from accessing PPC [4]. If accessing PPC is already challenging, end-of-life care for these patients can be also difficult. Therefore, this study is based on a recent mapping effort aimed at reflecting on the end-of-life care for children in Palliative Care in Brazil.

## Method

This study is a descriptive, cross-sectional, and online survey, based on a larger project developed by the Palliative Care Network (BPPCN), which is a collaborative effort developed at the Federal University of São Carlos. The BPPCN includes professionals affiliated with institutions across all Brazilian states, who are directly involved in the assistance, teaching, and research of PPC in Brazil. Therefore, a snowball sampling strategy was employed to reach as many participants as possible to complete the questionnaire.

This work represents a new sub-analysis derived from research that has already been the subject of other published studies [2, 4]. It employs a novel approach by incorporating variables not previously explored or by complementing existing discussions and results. The primary aim is to expand upon and enrich the conclusions previously presented.

As a convenience sample, invitations were sent via online platforms by BPPCN, such as email and WhatsApp, using a snowball strategy. The invitations were sent on different dates to representatives of palliative care services operating in Brazil that serve patients from birth to 21 years of age. Upon accepting the invitation, participants received a link to the questionnaire, which was hosted on Google Forms. Data collection occurred from February to May 2021. Duplicated responses were excluded, and only one representative per service was accepted.

The Checklist for Reporting of Survey Studies was used as the reporting guideline [6]. The questionnaire was created based on previous studies and included the following components [7–11]: (1) Identification and characterization of services, (2) Characterization of health professionals, (3) Access to opioid prescriptions, and (4) Education and research. Following a literature review, relevant publications for Brazil were included and questions were developed. Evaluation rounds of the questions were conducted by experts involved in this research. The questionnaire’s usability and functionality were tested among all researchers before distribution.

This study was approved by the Research Ethics Committee (CAAE 39915620.2.0000.5504), and all participants signed the Informed Consent Form. To guarantee anonymity, the questionnaires didn’t collect the respondent’s personal name, but only their institution’s information, and e-mail addresses. All IP addresses were checked for duplicates and only the survey coordinator had access to the complete questionnaires’ answers. Data were stored in a password-protected computer’s database. Descriptive analyses were performed on the online survey data using Stata version 13.0 (Stata Corp, L.C.) and a self-developed data-extraction template. Results are presented as frequencies, 95% confidence intervals (95% CI), graphs, and figures.

## Results

A total of 15 invitations were sent to participate in the research. Following these invitations, 97 completed questionnaires were received. Of these, all were fully completed, but 7 were excluded due to duplication. Thus, the final sample comprised 90 PPC services.

**Fig. 1:**
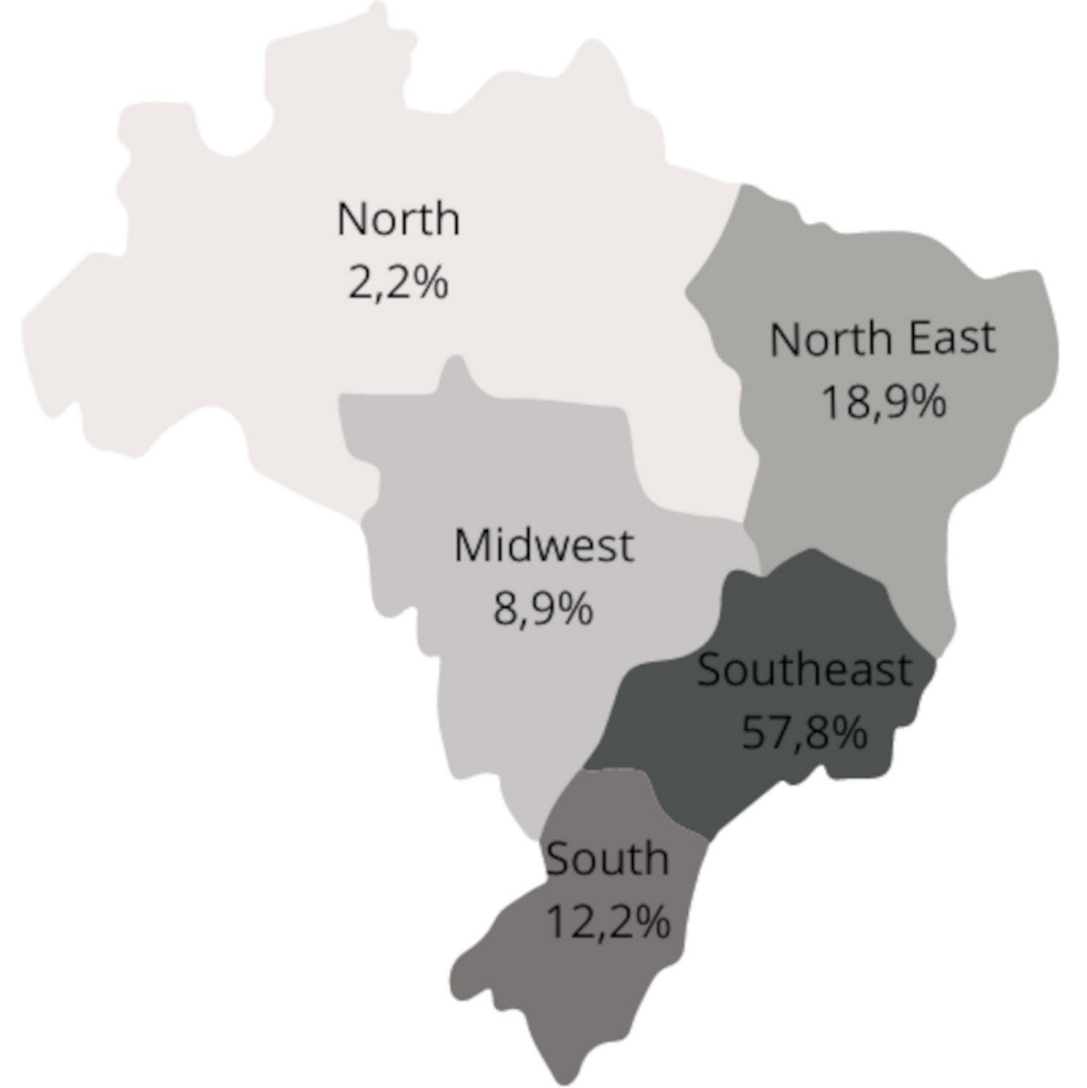
Distribution of pediatric palliative care services by region in Brazil, as shown in the map

The distribution of these services was uneven across the country. Of the 90 services, the state of São Paulo had the highest number, with 42.22% (38 services). By regions (see Fig. 1), the Southeast (population of 84.8 million inhabitants, 41.8% of total population) had the most services, accounting for 57.8% of the total (52 services), followed by the Northeast (population of 54.6 million inhabitants, 26.9% of total population), with 18.9% (17 services), the South (population of 29.9 million inhabitants, 14.7% of total population) with 12.2% (11 services), and the Central West (population of 16.3 million inhabitants, 8.02% of total population) with 8.9% (8 services). The North region (population of 17.3 million inhabitants, 8.5% of total population), despite being the largest in terms of territory, had the fewest registered services, representing only 2.2% of the total (2 services).

As shown in Table 1, the vast majority of services conduct PPC assessments through consultations (76.30%; 95% CI: 65.36–84.00%). Nearly half also provide care for children and adolescents on an outpatient basis (44.30%, 95% CI: 33.96–55.30%). Additionally, 33.00% (95% CI: 23.74% to 44, 05%) support hospitalized children without wards and intensive care units, 25.90% (95% CI: 16.94–35.83%) offer assessments via remote care, and only 20.60% (95% CI: 12.30–29.75%) provide home visits.

**Table 1:** Level of teams involvement in PPC assessments

Level of the team involvement	
Consultations	76.30%
Outpatient Care	44.30%
Hospitalized (Without wards and intensive care units)	33.00%
Remote Care	25.90%
Home Visits	20.60%

 Regarding the work team, 22 institutions (24.4%; 95% CI: 15.99–34.63%) reported having professionals who work exclusively in Palliative Care, while the remaining institutions (75.6%; 95% CI: 65.36–84.00%) have professionals who divide their time between PPC and other specialties. Regarding pediatrics, considering the institutions that provide PPC, 57 (63.33%; 95% CI: 52.51–73.24%) have professionals dedicated exclusively to pediatrics, whereas the remainder 33 (36.67%; 95% CI: 26.75–47.48%) have professionals who care for patients across various age groups. As shown in table 2, the average weekly commitment of the PPC team was more than 30 h in 5.56% of services(95% CI: 1.82–12.50%), between 10 and 30 h in 43.33% of services (95% CI: 32.91–54.19%), and less than 10 h in 51.11% of services (95% CI: 40.34–61.80%).

**Table 2:** Average weekly time dedicated by the team to Pediatric Palliative Care

Weekly commitment of PPC team	
More than 30 h	5.56%
Between 10 to 30 h	43.33%
Less than 10 h	51.11%

**Graph 1:**
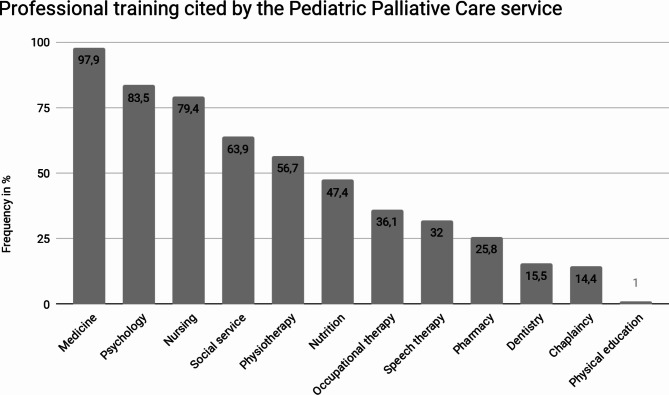
Percentage of professional training reported by representatives of the 90 pediatric palliative care teams

Regarding the professional training of team members, nearly all services have doctors (97.90%; CI 95%: 92.20–99.72%), the majority have psychologists (83.50%; CI 95%: 74.00–90.36%) and many have nurses (79.40%; 95% CI: 69.01–86.78%). Details on other aspects of professional training can be found in Graph 1.

Regarding pain management in pediatrics, the questionnaire assessed access to opioids within the services: 60.00% (95% CI: 49.13–70.18%) of the services reported that opioids were “fully accessible” for treating their patients’ pain, while 40.00% (95% CI: 29.81–50.86%) reported either “not having access” or “having difficulties accessing” opioids.

Regarding grief support, 36,70% (95% CI: 26.75–47.48%) of the services reported not providing any grief support for patients’ relatives, while 63,30% (95% CI: 52.51–73.24%) reported offering some form of grief support.

As shown in Table 3, regarding the types of support, the most frequently reported was the post-death telephone call, mentioned by 33 services (36.67%; 95% CI: 26.75–47.48%), followed by support groups for bereaved parents, mentioned by 22 services (24.4%; 95% CI: 15.99–34.63%) and post-mortem telegrams or letter, mentioned by 20,60% of services (95% CI: 12.30–29.75%).

**Table 3:** Types of support offered by the Pediatric Palliative Care services

Types of support	
Post-death telephone call	36.67%
Support groups for bereaved parents	24.40%
Post-mortem telegrams or letter	20.60%
Grief outpatient care	16.66%

The survey found that 37.78% of services in Brazil meet the minimum requirements, with some lacking key professionals such as doctors or nurses. As shown in table 4, different regions of Brazil also demonstrate differences in terms of minimum staffing.

**Table 4:** Percentage of services per region that meet the minimum requirements for a PPC team (at least one doctor, one nurse, one psychologist, one social worker, and physiotherapist)

Services that meet the minimum requirements for a PPC team	
Midwest	50.00%
North	0.00%
Northeast	29.41%
South	63.64%
Southeast	34.62%

## Discussion

In Brazil, there is limited data on end-of-life care during the death process of children in PPC. As a result, adequate and intensive management of symptoms in children and adolescents during their last days of life is compromised [12]. Therefore, this study provides an overview of essential aspects of proper end-of-life care, including available services, methods of care, team training and symptom control.

According to the World Health Organization, the number of children and adolescents eligible for pediatric palliative care globally can reach 21 million per year, with nearly 2.5 million dying each year in severe suffering. Brazil, a country of continental dimensions, has a limited number of PPC services relative to the existing demand. Moreover, the distribution of services is uneven, with regions, such as the North, having only two services for all children in that territory [7].

The majority of services provide care through consultations, with less than half providing outpatient follow-up and one-third providing care for hospitalized patients. Given that hospitals are the primary locations of death for children and adolescents in PPC, and the average duration of the “active phase of dying” is 25.2 h according to one study [12], it is likely that many patients may not receive adequate assistance at the end of life. This is because some patients do not have specialized teams that regularly accompany them in the death environment [12, 13].

Regarding PPC management, it is essential that services have a minimum team for PPC services to provide adequate care. This team should include at least one doctor, one nurse, one psychologist, one social worker, and one physiotherapist [1]. The research found that many services in Brazil do not meet this minimum team (only 37.78% of services meet the minimum requirements), with some lacking key professionals such as doctors or nurses. This difference highlights the regional disparities in Brazil when considering the relative percentage for each region (Table 4). While services in the South region have a percentage of 63.64% of services meeting the ideal minimum team requirements, services in the Northeast region show only 29.41%, and in the North region, no services meet the ideal minimum team requirements. It is noteworthy that palliative care presupposes working in a multidisciplinary team and providing care in multiple aspects. The vision of the different professional categories in relation to the PPC, as well as the complementary action, makes comprehensive care possible [1].

While the interdisciplinary team in PPC does not need to be exclusive dedicated, it is crucial for team members to have strong links and good interaction. The work should be multiprofessional, so that each specialty can collaborate together in the work of the interdisciplinary team [1].

The prevalence of symptoms in the last week of life for children undergoing PPC is high [12]. A 2021 study carried out by a multicenter group of PPC services highlighted that pain and dyspnea are frequently observed symptoms in pediatric patients nearing the end of life [13]. In this research, it is noteworthy that, although 60% of Brazilian services reported having free access to opioids, 40% still faced difficulties or lacked access to these medications. Since morphine is commonly used to manage pain and dyspnea in the end-of-life care in PPC [12], it is possible that symptom control may not be carried out adequately for children and adolescents [14].

Finally, concerning grief support for families, it is notable that a significant percentage of services do not provide any form of support. It is also noteworthy that the most common support mentioned by the services consists of post-death phone calls. This suggests that these situations are not being adequately handled in most places. With only about two-thirds of the services offering post-death support, none reported providing anticipatory grief support [15]. As understanding of grief support evolves with current theory and research on this topic, it is crucial for services to update their approaches. Effective grief support is essential for providing emotional assistance to parents, caregivers, and even healthy siblings [15].

## Conclusions

Reflecting on the mapping of PPC in Brazil, we can infer that end-of-life care for children and adolescents in the country is likely inadequate. There are both a limited number of services and significant regional disparities in their distribution in the territory, which may prevent patients from obtaining appropriate assistance. Most teams provide care through referrals and consists of a small number of professionals, making difficult the interdisciplinary approach and longitudinal monitoring that PPC requires for a global view of child’s and family’s needs. Moreover, difficulties in accessing opioids highlight a significant fragility in symptom management, especially for controlling pain and dyspnea. Additionally, over 30% of services lack grief support, which demonstrates the fragility of end-of-life care in pediatrics.

We conclude that there is a need for educational strategies, such as continuing education and inclusion of PPC in health courses, to improve care for children at the end-of-life, just as health service managers should also broaden their focus to address these issues more effectively.

## Limitations

While this study provides valuable insights into PPC in Brazil, several limitations must be acknowledged. These limitations may affect the conclusions and interpretation of the findings and should be considered.

### Sample size and generalizability

Although the sample size used in this study was adequate for the analysis, there were distortions related to the number of respondents in certain regions. For example, the North Region had a particularly low response rate. Future research should consider using a larger and more diverse sample to obtain more representative and comprehensive insights.

### Regional disparities

The significant regional disparities highlighted in the study may not account for all regional factors, such as local policies or economic conditions, which could influence the results. Further research should explore these regional differences in greater detail.

### Data Collection methods

The study used the snowball strategy for data collection, which can cause some distortions related to sampling bias (problems related to homogeneity of the sampling), dependence on initial contacts (the quality and diversity depends on the initial participants), and unpredictability of sample growth.

In conclusion, this study contributes valuable insights to the study of PPC in Brazil, however these limitations should be considered when interpreting the results. Addressing these limitations in future research can help to improve the study in the area and provide better results in the future.

## Electronic supplementary material

Below is the link to the electronic supplementary material.

Supplementary Material 1

Supplementary Material 2
